# Safeguarding early nutrition

**DOI:** 10.1016/j.eclinm.2023.102186

**Published:** 2023-08-22

**Authors:** eClinicalMedicine

Early nutrition has profound effects on child's growth, development, and overall wellbeing. Nutrition during the early years of life should support children's rapid growth and be tailored to their specific needs. Nutritional deficiencies during this crucial window can lead to irreversible cognitive impairment and physical abnormalities, such as stunted growth and rickets, underscoring the urgency of getting early nutrition right.^1^

After birth, one of the healthiest ways to promote optimal early nutrition is breastfeeding, which is recommended for at least 6 months to ensure optimal growth, if it is feasible for the mother. Several factors can influence mother's decision whether to breastfeed or formula feed their baby including medical considerations, individual preferences, and personal circumstances. During the first week of August each year, the World Breastfeeding Week is celebrated to promote breastfeeding worldwide and to raise awareness of its benefits. The clearest being the protection against infections and the reduced risk of sudden infant death syndrome, allergy, asthma, type 1 diabetes, celiac disease, and obesity.^2^

The role of early nutrition in influencing lifelong health outcomes gained prominence due to the research conducted by David Barker and his colleagues, who observed that an individual's likelihood of developing cardiometabolic diseases in adulthood was connected to their nutritional status during intrauterine development and birthweight.^3^

Recent evidence also indicates that during the first 1000 days, from conception to the child's second birthday, key developmental processes occur that lay the foundation for various physiological and cognitive functions. Adequate nutrition, particularly in terms of macronutrients, micronutrients, and other essential factors, is a cornerstone for the healthy growth and development of organs, tissues, and neural networks.^4^ During these early years the development of primary systems and neurobehavioral processes including learning and memory formation, speed of processing and reward mechanisms take place and are at greatest risk of nutrient perturbations.^5^

Of note, malnutrition includes both undernutrition and excessive calorie intake, often at the expense of other essential nutrients. Both forms of malnutrition affect neurodevelopment, and they can coexist in an individual.

Malnutrition, particularly excessive calorie intake is also responsible for childhood obesity, which is a growing concern worldwide. WHO reports that overweight and obesity affects more than 340 million children and adolescents—with 39 million children under the age of 5 years with overweight or obesity in 2020. These children are facing greater risk of developing early type 2 diabetes, fatty liver, and cardiovascular complications. Although complex and multifactorial, the pathophysiology of obesity recognises the central role of poor nutrition, coupled with a sedentary lifestyle. Reducing the intake of sugary snacks and beverage and processed foods is crucial in maintaining a healthy weight,^6^ although there might be several contributing factors.

Last month WHO released new guidelines presenting the latest scientific evidence on the role of fats^7^ and carbohydrates^8^ in a healthy diet, particularly in children. The guidelines comprise specific recommendations aimed at decreasing excessive weight gain and the risk of non-communicable diseases, such as cardiometabolic diseases and cancer. Particular attention was paid to the role of saturated fats in cardiovascular disease development and insulin resistance. The document offers guidance to replace unhealthy saturated fats and trans-fatty acids, which are mainly found in animal and ultra-processed products. Ultra-processed foods pose a considerable challenge because they are often convenient and have a long shelf life, but contain a high number of additives, preservatives, sugars, unhealthy fats, and artificial ingredients and usually lack essential nutrients such as vitamins, minerals, and dietary fibres.

Despite the growing concerns over the potential harmful health effects of ultra-processed foods, these foods are aggressively marketed to encourage consumption and are replacing traditional dietary habits centred around fresh and minimally processed foods. Earlier this year, WHO Regional Office for Europe published a document aimed at protecting children from marketing that promotes unhealthy food and sugary beverages.^9^ The published set of nutritional criteria can help to determine whether food products are suitable to be marketed to children and young people, and whether baby foods are appropriate for infants younger than 3 years.

This guidance is particularly relevant for children who might develop unhealthy eating habit of favouring ultra-processed foods and altered taste perception, which can potentially lead to a lifelong preference for less nutritious and unhealthy options as the eating habits formed in childhood often persist into adulthood.^10^

Family meals could offer an opportunity for parents to model good eating behaviours and foster positive associations with nutritious foods. However, a meta-analysis published in eClinicalMedicine showed only a weak to moderate association between parent and child dietary choices,^(ref)^ highlighting the role of personal preference and overall environment for dietary habits and making it crucial to establish comprehensive guidelines on food products that can be marketed to children.

Harmful marketing is not the only challenge we face. The prevalence of unhealthy foods will only get worse with the increasing cost of living and the difficulty for many families to afford healthy and nutritious food and thus the reliance on ultra-processed foods to feed their children. Restricting consumption of ultra-processed foods is only feasible if policies are put in place to financially support healthier options, especially for low-income families and school meal programmes.

This approach might come with a hefty price tag, but investing in early nutrition has far-reaching implications, extending beyond individual health to broader social and economic outcomes.

Children who receive healthy diets early in life are more likely to perform well academically.^1^^,^^11^ Education, in turn, is a key factor in breaking the cycle of poverty and empowering young people. By building a strong foundation through early nutrition, we can address, at least in part, the economic disparities and foster a more equitable society.

Governments, health care providers, and communities must work together to ensure that every child has access to quality nutrition during the crucial period of early development and beyond. Policies that prioritise maternal and child nutrition, promote and support breastfeeding, and endorse nutrition education can achieve far-reaching health benefits.

The importance of early nutrition transcends individuals, and it is a keystone for societal progress to foster a generation that is not just physically healthy, but able to reach its full potential.
